# Multiple Mechanisms of Photoreceptor Spectral Tuning in *Heliconius* Butterflies

**DOI:** 10.1093/molbev/msac067

**Published:** 2022-03-28

**Authors:** Kyle J. McCulloch, Aide Macias-Muñoz, Ali Mortazavi, Adriana D. Briscoe

**Affiliations:** 1 Department of Ecology and Evolutionary Biology, University of California, Irvine, Irvine, CA, USA; 2 Department of Ecology, Evolution, and Behavior, University of Minnesota, St Paul, MN, USA; 3 Department of Ecology, Evolution, and Marine Biology, University of California, Santa Barbara, Santa Barbara, CA, USA; 4 Department of Developmental and Cell Biology, University of California, Irvine, Irvine, CA, USA

**Keywords:** ultraviolet, color vision, photoreceptor, short wavelength opsin, Lepidoptera, coexpression

## Abstract

The evolution of color vision is often studied through the lens of receptor gain relative to an ancestor with fewer spectral classes of photoreceptor. For instance, in *Heliconius* butterflies, a genus-specific *UVRh* opsin duplication led to the evolution of UV color discrimination in *Heliconius erato* females, a rare trait among butterflies. However, color vision evolution is not well understood in the context of loss. In *Heliconius melpomene* and *Heliconius ismenius* lineages, the UV2 receptor subtype has been lost, which limits female color vision in shorter wavelengths. Here, we compare the visual systems of butterflies that have either retained or lost the UV2 photoreceptor using intracellular recordings, ATAC-seq, and antibody staining. We identify several ways these butterflies modulate their color vision. In *H. melpomene,* chromatin reorganization has downregulated an otherwise intact *UVRh2* gene, whereas in *H. ismenius*, pseudogenization has led to the truncation of UVRh2. In species that lack the UV2 receptor, the peak sensitivity of the remaining UV1 photoreceptor cell is shifted to longer wavelengths. Across *Heliconius*, we identify the widespread use of filtering pigments and co-expression of two opsins in the same photoreceptor cells. Multiple mechanisms of spectral tuning, including the molecular evolution of blue opsins, have led to the divergence of receptor sensitivities between species. The diversity of photoreceptor and ommatidial subtypes between species suggests that *Heliconius* visual systems are under varying selection pressures for color discrimination. Modulating the wavelengths of peak sensitivities of both the blue- and remaining UV-sensitive photoreceptor cells suggests that *Heliconius* species may have compensated for UV receptor loss.

## Introduction

The visual systems of arthropods and vertebrates have evolved to discriminate many distinct colors, complementing the salient visual signals found in the animal's visual space (Briscoe and Chittka 2001; Osorio and Vorobyev 2005; Futahashi et al. 2015). In animals that inhabit and sense colorful environments, the evolution of new spectral channels—mainly achieved through duplication of opsin genes followed by sequence divergence to generate novel photoreceptor cell types—has been the focus of most research (Cronin et al. 1996; Briscoe and Chittka 2001; Parry et al. 2005; Frentiu et al. 2007; Osorio and Vorobyev 2008; Thoen et al. 2014; Chen et al. 2016). Opsin proteins are covalently bound to a vitamin A-derived chromophore to make rhodopsin, the visual pigment responsible for the detection of light in animal photoreceptor cells (Palczewski et al. 2000). Among opsin paralogs, amino acid substitutions that interact with the chromophore may shift the absorption spectrum of a particular rhodopsin (Arshavsky et al. 2002; Shichida and Matsuyama 2009; Bloch 2016). Other ways of modifying a photoreceptor neuron's sensitivity to light include the addition of photostable filtering pigments and co-expression of multiple opsins in a single cell (Arikawa et al. 1999, 2003; Wakakuwa et al. 2004; Knott et al. 2010; Dalton et al. 2014; Satoh et al. 2017; Vöcking et al. 2017).

Despite the apparent ease with which opsins duplicate, most vertebrates have four or fewer spectral channels, perhaps because of the increasing complexity of the neural wiring required to add spectrally opponent channels to the nervous system (Rushton 1972; Barlow 1981; Buchsbaum and Gottschalk 1983; Thornton and Pugh 1983; Osorio 1986; Dacey 1999; Kelber and Osorio 2010). Given constraints on adding more spectral types of photoreceptor, such additions may not always be adaptive. Shifting selection pressures or drift might result in the loss of spectral channels, which could be beneficial, even in a colorful world. The loss of visual capability is generally studied in the context of low-light environments where color vision is no longer necessary, such as in shifts to nocturnality, caves, or deep ocean (Crandall and Hillis 1997; Friedrich et al. 2011). We decided to address the molecular and physiological evolution of visual systems of *Heliconius* butterflies with a known loss of a UV-sensitive cell despite no apparent loss in their color sensing demands.

**Fig. 1.**
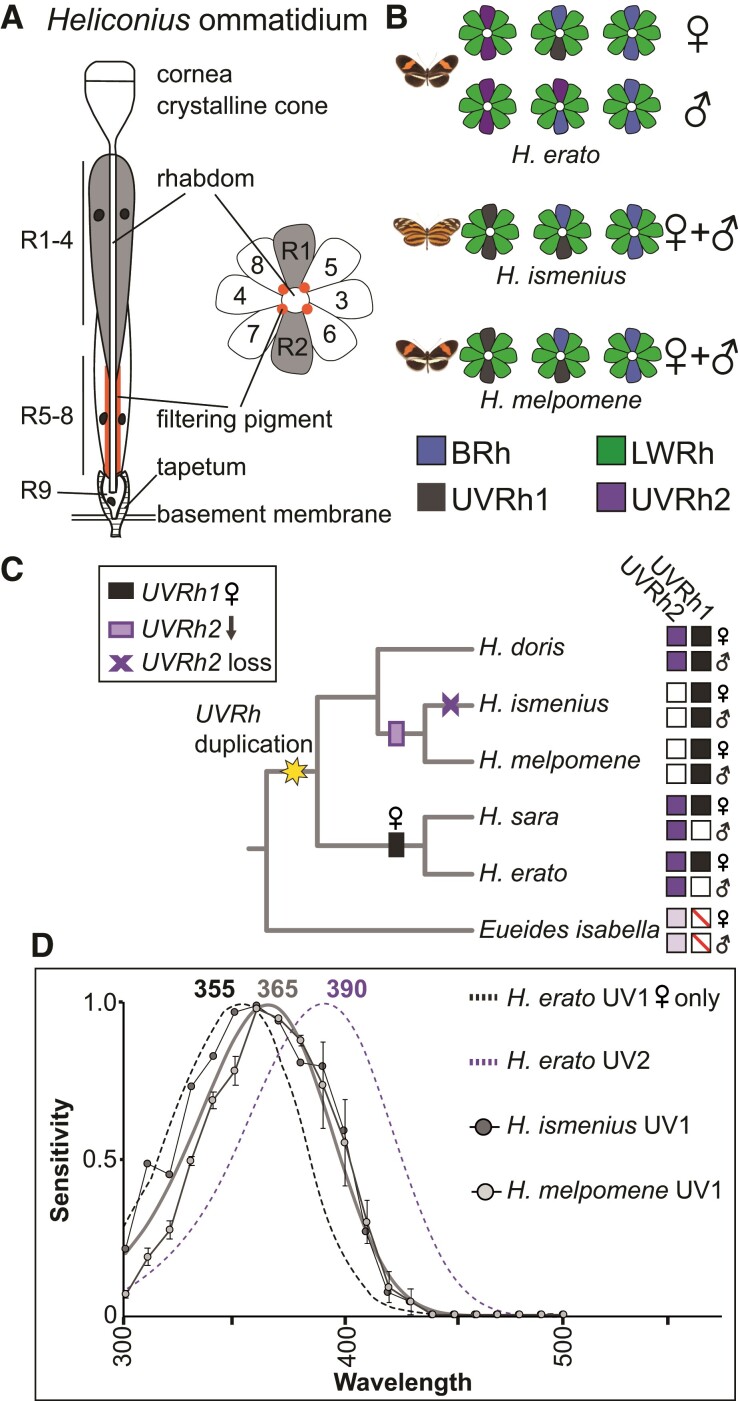
Heliconius eye anatomy, evolutionary history of UV opsin gene duplication and loss, and UV photoreceptor spectral sensitivities. (*A*) Overview of *Heliconius* ommatidium structure. Light enters and is focused through the cornea and crystalline lens and passes through the rhabdom. R1–4 cells contribute more distally to the rhabdom, whereas R5–8 cells and filtering pigment (when present) contribute more proximally. The tiny R9 cell sits at the proximal end of the rhabdom. Photoreceptor cell axons project through the basement membrane to the optic lobe. In transverse sections, R1 and R2 cells sit opposite each other, whereas diagonal R5–8 have red pigment next to the rhabdom from ∼320 to 480 µm below the cornea (Zaccardi et al. 2006). The density of this pigment is heterogeneous between ommatidia across the eye, producing yellow- and red-reflecting adjacent ommatidia. (*B*) Cross-sections of ommatidial types found in the compound eyes of *Heliconius erato*, *Heliconius melpomene*, and *Heliconius ismenius*, based on previous antibody staining (see new results with expanded ommatidial types in fig. 5 and supplementary fig. S3, Supplementary Material online) (McCulloch et al. 2017). Ommatidial types are classified by the combination of short-wavelength opsins expressed in R1/R2. (*C*) Simplified phylogeny of species representing major *Heliconius* clades with *Eueides* outgroup lacking the *UVRh* duplication. Character mapping is based on the maximum likelihood ancestral state reconstruction of UV opsin immunohistochemistry in both sexes of 13 *Heliconius* species and 1 *Eueides* species and ancestral state reconstruction of UV loss-of-function mutations in 26 *Heliconius* and 1 *Eueides* species, as shown in fig. S3 of McCulloch et al. (2017) and reproduced in supplementary fig. S2, Supplementary Material online. The *Heliconius UVRh* duplication occurred at the base of the genus (Briscoe et al. 2010), giving rise to functional UVRh1 and UVRh2 opsins. Subsequent loss of mRNA expression is mapped across the phylogeny with white squares. *UVRh2* mRNA downregulation was followed by pseudogenization in the silvaniform clade (purple x) but not in the *H. melpomene* clade (purple rectangle). Female-limited UVRh1 expression in the *erato* and *sara* clades is indicated with a black rectangle. Protein expression presence or absence is shown to the right for both sexes, presence with filled-in purple or black squares, absence with empty squares. *Eueides isabella* only has one *UVRh* locus, indicated by light shading and red line through box; its *UVRh* genomic location is the same as that of *UVRh2* in *Heliconius,* suggesting that this is the ancestral gene locus. (*D*) The measured UV1 photoreceptor cell sensitivities from *H. ismenius* (silvaniform) (dark gray circles) and *H. melpomene* (light gray circles), and approximate template fit for both species (within margin of error, supplementary fig. S1 and table S4, Supplementary Material online) are shown. For comparison, rhodopsin absorbance templates derived from measured spectral sensitivities of *H. erato* UV1 and UV2 photoreceptor cells (McCulloch et al. 2016a) are indicated by black and purple dotted lines, respectively. *Heliconius erato* males and females have UV2 cells with peak sensitivity at 390 nm, while only females also have the UV1 cell with a peak at 355 nm. *Heliconius ismenius* (*n* = 1) and *H. melpomene* (*n* = 3) UV1 cells peak at the longer wavelength of 365 nm compared with *H. erato.* Both species lack UVRh2 protein expression.

Butterflies in the genus *Heliconius* have superb color vision from UV to red wavelengths (Crane 1955; Swihart and Swihart 1970; Zaccardi et al. 2006; Finkbeiner and Briscoe 2021). The morphological basis of *Heliconius* color vision is the compound eye, which is similar in structure to other butterflies. The eye is a retinal mosaic of thousands of unit eyes, called ommatidia. Each ommatidium is a long tube of nine photoreceptor cells that project axons to the optic lobe (fig. 1*A*). The photoreceptor cells (R1–9) project microvilli packed with rhodopsin into the center of the ommatidium, forming a fused fiberoptic-like structure called the rhabdom. In transverse sections, the R1–8 cells in a single ommatidium are arranged like petals on a flower with the rhabdom in the center (fig. 1*A*). Light is focused through the cornea and crystalline cone and channeled through the rhabdom where it is absorbed by rhodopsins of each photoreceptor cell type. R1 and R2 cells are typically variable short-wavelength photoreceptor cells (BRh- or UVRh-expressing), involved in color vision (Borst et al. 2010; Schnaitmann et al. 2018). The R3–8 cells express LWRh, are green-sensitive, and are involved in motion and contrast vision, though a subset may also contribute to color processing (Chen et al. 2020). Some diagonal R5–8 cells have a red pigment next to the proximal rhabdom, a filtering pigment responsible for yellow-to-red color vision in *Heliconius* (fig. 1*A*) (Zaccardi et al. 2006).

The genus *Heliconius* has undergone an adaptive radiation throughout the Neotropics, which is most visibly evident in their spectacular diversity of aposematic wing patterns that also serve as sexual signals (Smiley 1978; Brown 1981; Williams and Gilbert 1981; Jiggins et al. 2001; Estrada and Jiggins 2002; Joron et al. 2006; Kronforst et al. 2007; Estrada and Gilbert 2010; Hill et al. 2013; Merrill et al. 2015, 2019). This complex visual ecology within the genus is reflected in significant variation among the adult compound eye retinal mosaics found across species. Much of this eye diversity stems from a UV opsin (*UVRh*) duplication that occurred at the base of the genus, leading to two distinct UV-sensitive R1 and R2 photoreceptor cell types (Briscoe et al. 2010). Subsequently, lineage-specific variation in the spatial expression of particular UV-sensitive R1 and R2 cell types gave rise to at least three forms of sexual dimorphism. In *Heliconius doris*, both sexes express UVRh1 and UVRh2 opsins (McCulloch et al. 2017). In the sexually dimorphic visual system of *Heliconius erato*, females express UVRh1 and UVRh2 opsins, whereas males only express UVRh2 (fig. 1*B*). Intracellular recordings and behavioral experiments showed that *H. erato* females have two physiologically distinct UV photoreceptor cell types and that these conferred UV color vision in females only (McCulloch et al. 2016a; Finkbeiner and Briscoe 2021). Differences in the retinal mosaics across sexes and species showcase *Heliconius* as an evolutionary model to study incipient visual system divergence (McCulloch et al. 2017).

In two other *Heliconius* clades, protein expression of one of the UV opsins was lost: the tiger-wing silvaniform clade (e.g., *Heliconius ismenius*, *H. hecale*) and the *melpomene/cydno* clade do not express UVRh2 in their retinal mosaics (McCulloch et al. 2017) (fig. 1*B* and *C*). In the silvaniforms, *UVRh2* is currently undergoing pseudogenization and little to no mRNA expression is detected (see table S2 in McCulloch et al. 2017). Independent loss-of-function mutations have accumulated among silvaniform species, suggesting that this process began in parallel after the split from the *melpomene/cydno* sister clade (McCulloch et al. 2017). Meanwhile in *melpomene/cydno* species, expression of full-length *UVRh2* is low but not absent, even though protein expression is missing from the eye. Consistent with a loss of UV2 R1/R2 cells, our behavioral experiments indicate that both male and female *H. melpomene* are unable to discriminate between 380 and 390 nm light (Finkbeiner and Briscoe 2021). Thus, although both silvaniform and *melpomene/cydno* clades lack a UV2 photoreceptor, differences may exist in the timing and manner in which these lineages have downregulated *UVRh2,* as well as other potential changes to visual systems. Our previous work suggested that the *Heliconius* visual system (as exemplified by *H. erato*), likely compensated after gene duplication and neofunctionalization of *UVRh2* via spectral tuning to accommodate a new color channel (McCulloch et al. 2016a; Finkbeiner and Briscoe 2021). Is the *loss* of this same receptor in some lineages correlated with the spectral tuning of their remaining photoreceptors, suggesting subsequent compensation? Since the loss of this UV cell type could mean the loss of UV color vision, as observed in *H. melpomene*, we were interested in comparing the molecular, cellular, and physiological basis of this loss between these species.

To better understand the mechanisms of color vision evolution in these closely related species, we investigated opsin expression and photoreceptor cell function in *H. melpomene* and *H. ismenius.* We compared our new findings with previously published *H. erato* data, giving a unique perspective on photoreceptor cell evolution after the loss of a recently gained opsin-based receptor (∼4.5 Mya; Kozak et al. 2015). To explain discrepancies between gene expression and protein expression, as well as to explore potential mechanisms of *UVRh2* loss, we investigated chromatin regulation in the two *UVRh* loci in *H. melpomene.* Here, we find that *H. melpomene* and *H. ismenius* have used a variety of spectral tuning mechanisms to modify photoreceptor sensitivities, resulting in distinct suites of photoreceptor subtypes between each other and *H. erato.* We also identify co-expression of blue and green opsins and a novel broadband cell type in these species. Together we show how several tools for spectral tuning allow for rapid shifts in *Heliconius* visual systems following UV2 receptor loss.

## Results

### UV Photoreceptor Spectral Tuning and *cis*-Regulation in *Heliconius UVRh* Loci

We first asked whether UVRh2 loss might alter UV photoreceptor cell spectral sensitivity in *H. ismenius* and *H. melpomene* relative to *H. erato.* Both *H. ismenius* and *H. melpomene* lack a UVRh2-expressing cell (fig. 1*C*). This suggests a loss in these two species of the 390 nm cell type found in *H. erato*. Behavioral tests of *H. melpomene* confirm adult males and females are unable to discriminate 380 nm from 390 nm light while they do retain the ability to discriminate 400 nm from 436 nm light (Finkbeiner and Briscoe 2021). Intracellular recordings reported here reveal a single UV-sensitive R1/R2 photoreceptor cell type in both *H. melpomene* and *H. ismenius*, with peak sensitivity (*λ*_max_) = 365 nm (fig. 1*D*), consistent with behavioral experiments. We were not able to record from a UV cell in the *H. ismenius* male or the *H. melpomene* female. Due to the lower proportion of UV cells in the eyes of these species and limitations in our recording setup, it is technically challenging to record from UV cells through random sampling. As reported by us, counts of antibody staining on complete retinas reveal roughly 75% of the cells available for recording in all *Heliconius* retinas are LW-sensitive, while SW-sensitive cell populations differ by lineage. In silvaniform and *melpomene/cydno* clades, roughly 20% of cells are blue-sensitive, while only 5% of cells in the retina are UV1 cells, and these do not differ significantly in number by sex or across species (McCulloch et al. 2017). Given this technical challenge, and the fact that the gene sequence and protein expression patterns are quite similar and that the *H. ismenius* female receptor nearly perfectly matches that of the *H. melpomene* male receptor, it is likely that the sensitivities of UV1 receptors are similar between sexes and species. This UV1 receptor peak is shifted 10 nm toward longer wavelengths compared with the *H. erato* UV1 cell type with *λ*_max_ = 355 nm.

**Fig. 2.**
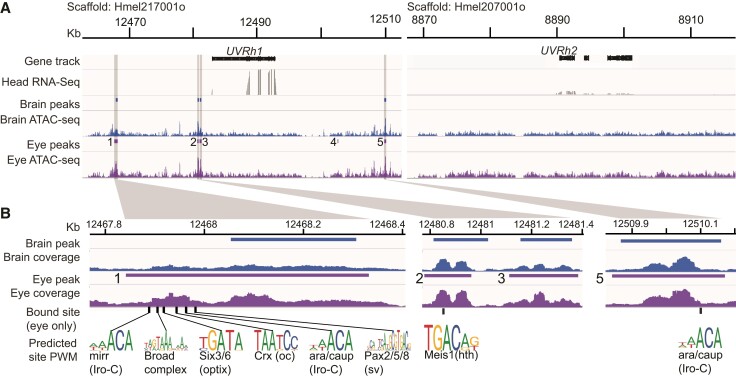
Chromatin state of *UVRh1* and *UVRh2* in adult *H. melpomene* eye and brain. (*A*) Fifty kilobase pair regions surrounding *H. melpomene UVRh1* and *UVRh2* loci showing ATAC-seq reads and predicted peaks of open chromatin for the brain (blue) and eye (purple) samples, and whole-head RNA-seq reads (dark gray). “Head RNA-seq” shows mRNA sequencing read coverage from whole-head samples, “Brain” and “Eye ATAC-seq” show ATAC sequencing read coverage from each tissue. Five significant peaks of open chromatin are found near *UVRh1* in eye samples and only four of these are significant in the brain sample. The *UVRh2* locus is much more closed and no significant peaks were detected in either ATAC-seq sample. RNA-seq coverage is significantly lower at the *UVRh2* locus compared with *UVRh1* (McCulloch et al. 2017). (*B*) Zoomed-in peaks (location of lighter gray columns in *A*) showing selected vision-related transcription factor binding sites predicted to be bound by TOBIAS analysis in the *UVRh1* locus in eyes only. Peak 4 did not have any bound sites. No brain peaks have bound transcription factors (see supplementary table S2, Supplementary Material online for bound/unbound transcription factor binding sites in each peak). A subset of bound sites for known eye-related transcription factors are highlighted and the position weight matrices for each are shown (from JASPAR). mirr, mirror; ara/caup, araucan/caupolican; hth, homothorax; oc, ocelliless (or otd); sv, shaven.

The loss of the UV2 cell results in a significant change in the sensory capability of *H. melpomene* (and presumably in *H. ismenius*): loss of UV color vision. To understand potential mechanisms for this loss, we further investigated the regulation of *UVRh1* and *UVRh2.* Previously, we showed that *UVRh2* is expressed at low levels in *H. melpomene* heads despite no protein expression in the compound eye (McCulloch et al. 2017). To determine whether this is a post-transcriptional modification, if UVRh2 is expressed elsewhere, or whether chromatin regulation can be attributed to differences in *UVRh* expression, we used Assay for Transposase-Accessible Chromatin using sequencing (ATAC-seq). We targeted *H. melpomene* for sequencing due to its well-annotated genome (*Heliconius* Genome Consortium 2012; Davey et al. 2016). We generated ATAC-seq libraries from the brain and compound eye photoreceptor cells of two *H. melpomene* adults. Each library had on average ∼35 million reads and ∼92% of the reads mapped and paired properly (supplementary table S1, Supplementary Material online). We used MACS2 to identify ATAC-seq read peaks denoting open chromatin and merged tissue replicates to find consensus peaks. We found that the *UVRh1* locus is indeed more open than *UVRh2* indicating chromatin around *UVRh1* is more available for transcriptional regulation. We identified three peaks upstream of *UVRh1* in both the brain and eye, and two peaks downstream, one of which is only found in the eye but not the brain (fig. 2*A*). There were no peaks near *UVRh2* in either eye or brain samples, indicative of a more closed chromatin state in this region (fig. 2*A*). This suggests *UVRh2* is not expressed elsewhere in the brain (although *UVRh1* could be), and that chromatin architecture limits transcription at the *UVRh2* locus. That UVRh2 protein is never detected in the eye suggests the transcript is being degraded before translation or the peptide is being degraded as it is being made. Together our data suggest that despite an intact transcript, multiple molecular mechanisms are being employed to block a UVRh2-expressing receptor subtype in *H. melpomene*.

Next, we sought to identify potential transcription factors regulating *UVRh1.* While chromatin could be open, transcription factors may not be currently bound and regulating transcription. We looked for evidence of bound transcription factors at known binding motifs using TOBIAS (Bentsen et al. 2020) in the *UVRh1* peaks in both the eye and brain (fig. 2*A* and *B*, supplementary table S2, Supplementary Material online). TOBIAS classifies sites as either in the bound or unbound state (supplementary table S2, Supplementary Material online). Our data show that transcription factors are bound in the eye but not in any peaks in the brain, indicating more transcriptional activity in the eye. In the eye, peaks 1, 2, and 5 are bound by transcription factors. Eye peak 1 has several bound sites for transcription factors known to be involved in eye development. We identified bound sites for two members of the retinal determination gene network, Pax2/5/8 and Optix, which are classical regulators of eye development in both *Drosophila* and vertebrates (fig. 2*B*, supplementary table S2, Supplementary Material online) (Fu and Noll 1997; Kumar 2009). We also detected binding for the two TALE-class homeodomain proteins of the Iroquois complex (Araucan/Caupolican and Mirror), which are important in eye imaginal disc development as well as adult regulators of rhodopsin expression (Mazzoni et al. 2008). Other bound sites include ones for Ocelliless/Crx, which is a paired domain homeobox protein and known regulator of rhodopsin in *Drosophila* (Tahayato et al. 2003), and the Broad-complex zinc finger, which is required for morphogenetic furrow progression and proper R8 specification in the compound eye (Brennan et al. 2001). A bound Maf subfamily site in eye peak 2 is predicted to be similar to Neural-retina-specific leucine zipper protein, which has been shown in mice to directly bind to the rod-opsin promoter and is required for rod differentiation, though its role in *Drosophila* eye development is unknown (Mears et al. 2001). This peak also contains a bound site for the TALE-class homeodomain protein Homothorax/Meis1, involved in delimiting the eye field and in differentiation of dorsal rim area photoreceptors in *Drosophila* (fig. 2*C*) (Pai et al. 1998; Wernet et al. 2003). Eye peak 5 contains a second bound site for Iroquois complex (Araucan/Capolican) transcription factors. Other bound sites include for NR2F1 and RXR in peak 1, CREB, ATF6 in peak 2, and multiple C2H2 zinc finger and bHLH factors in peak 5. These are all general transcriptional regulators but many have also been identified as key regulators in visual system development (Kumar et al. 2004; Chen et al. 2017; Cvekl and Zhang 2017; Klein et al. 2017; Kroeger et al. 2021). Although *UVRh1* chromatin is open in both the eye and brain, evidence that this locus is transcriptionally active via bound transcription factors is only found in the eye. In summary, we identified the potential binding of *H. melpomene* homologs of several transcription factors (Shaven/Pax2/5/8, Optix/Six3/6, Ocelliless/Crx, Homothorax/Meis1, Broad complex, Araucan/Caupolican, and Mirror) involved in *Drosophila* eye development. These bound transcription factors, a subset of known eye development genes, are good candidates for an undescribed function: adult UV1 cell maintenance.

### Spectral Tuning in LWRh- and BRh-Expressing Cells in *H. melpomene* and *H. ismenius*

**Fig. 3.**
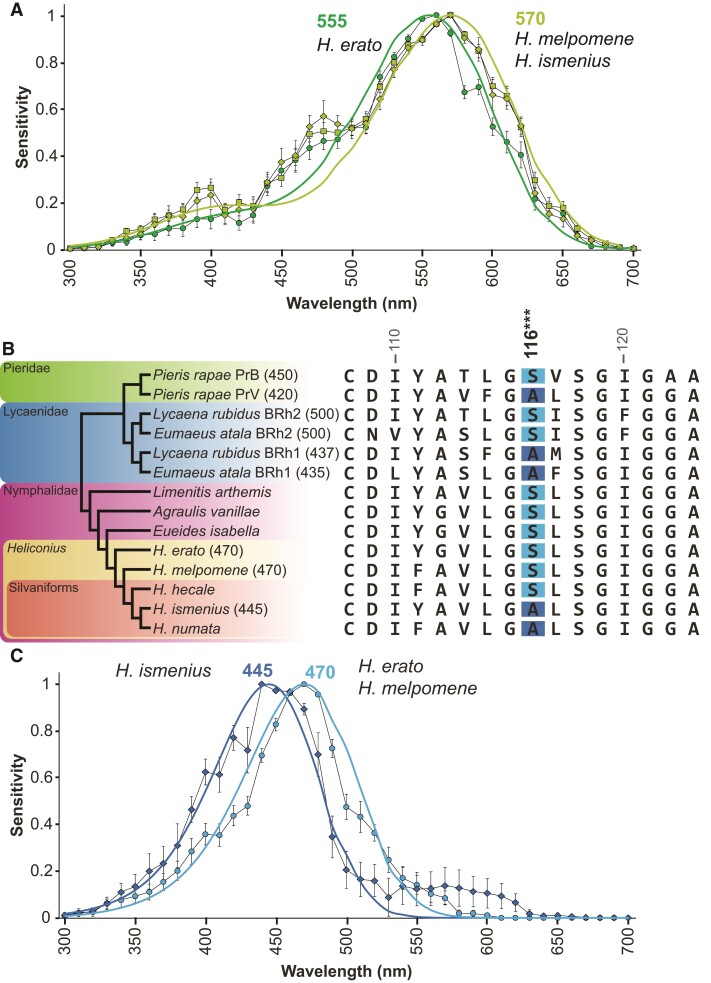
Opsin-based spectral tuning of long- and blue-wavelength photoreceptor cell sensitivities. (*A*) Long-wavelength photoreceptor spectral sensitivities of *H. erato* (green circles, *λ*_max_ = 555 nm, *n* = 19) (McCulloch et al. 2016a), *H. ismenius* (chartreuse diamonds, *λ*_max_ = 570 nm, *n* = 11), and *H. melpomene* (chartreuse squares, *λ*_max_ = 570 nm, *n* = 22) peak in the green range as fitted by the rhodopsin nomogram (Stavenga et al. 1993; Stavenga 2010). (*B*) Species phylogeny of butterflies (left) with known opsin peak absorbances (in parentheses) based on expression in HEK293 cells (Wakakuwa et al. 2010; Frentiu et al. 2015; Liénard et al. 2021), retinal densitometry (Bernard and Remington 1991) or intracellular recordings (present study; McCulloch et al. 2016a). Blue opsin alignment in the region around the spectral tuning site 116. In distantly related butterflies (*Pieris, Eumaeus*), a Ser116Ala substitution causes a blue-shift in blue rhodopsin absorbance in site-directed mutagenesis experiments (Wakakuwa et al. 2010; Liénard et al. 2021). *Heliconius* and other Heliconiini have a single blue opsin. *Heliconius erato, H. melpomene,* and *H. hecale* have Ser at position 116, whereas *H. ismenius* and *H. numata* have Ala. Numbering is relative to squid rhodopsin. (*C*) Averaged spectral sensitivities of *Heliconius* blue-sensitive cells. The *H. ismenius* blue-sensitive photoreceptor peak is blue-shifted (dark blue diamonds, *λ*_max_ = 445 nm, *n* = 4) relative to *H. melpomene* (light blue circles, *λ*_max_ = 470 nm, *n* = 7) and *H. erato* (data shown in McCulloch et al. 2016a, *λ*_max_ = 470 nm, *n* = 11). Dark and light blue lines indicate fits to 445 and 470 nm rhodopsin templates, respectively. Sensitivities averaged across sexes.

We next investigated if the visual systems of *H. melpomene* and *H. ismenius* might have compensated for the loss of the 390 nm photoreceptor cell by tuning other spectral classes of photoreceptor. *Heliconius erato* has a green-sensitive photoreceptor that peaks at *λ*_max_ = 555 nm, while in *H. melpomene* and *H. ismenius*, we find that this cell peaks at *λ*_max_ = 570 nm, a shift toward longer wavelengths (fig. 3*A*). As both green cells’ sensitivities are well fit with the rhodopsin nomogram (supplementary fig. S1 and tables S3 and S4, Supplementary Material online) (Stavenga et al. 1993), amino acid substitution in the green opsin alone is likely sufficient to explain the differences in peak sensitivity.

We asked whether middle-wavelength differences might exist in the absence of the UV2 R1 and R2 cell. The *H. erato* blue cell is long-wavelength shifted (*λ*_max_ = 470 nm) compared with most insect blue photoreceptors (∼450 nm; Briscoe and Chittka 2001; see also van der Kooi et al. 2021), perhaps to accommodate the presence of the 390 nm receptor. We therefore hypothesized that the blue receptor might be shifted toward shorter wavelengths in *Heliconius* species lacking the 390 nm receptor, to better discriminate colors in the blue/violet range. *Pieris rapae, Eumaeus atala, Lycaena rubidus*, and other butterflies have duplicated blue opsins with distinct peak wavelengths of absorbance. Functional work in *Pieris, Limenitis*, and *Eumaeus* has shown a number of spectral tuning sites responsible for shifts in the wavelength of peak absorbance (Wakakuwa et al. 2010; Frentiu et al. 2015; Liénard et al. 2021); however, all *Heliconius* sequences are invariant at all of these sites except for one. In *P. rapae* and *E. atala,* Ser116Ala mutation is responsible for a ∼5–13 nm blue-shift in the peak absorbance of blue rhodopsins expressed in cell culture (Wakakuwa et al. 2010; Liénard et al. 2021). The substitution at site 116 is also present in the independently duplicated blue opsins of *L. rubidus,* which encode 437 and 500 nm rhodopsins (fig. 3*B*) (Sison-Mangus et al. 2006; Wakakuwa et al. 2010). We found that *H. erato* and *H. melpomene* have serine in position 116 while the silvaniforms *H. ismenius* and *Heliconius numata* have substituted alanine (fig. 3*B*). Other closely related *Heliconius* species including silvaniforms (*H. hecale* shown fig. 3*B*) have a serine at this position. The phylogenetic placement of the alanine substitution in *H. ismenius* and *H. numata* suggests that this has recently evolved—independent of mutations in pierid and lycaenid butterflies—within silvaniforms after the lineage split from the *melpomene/cydno* clade. Indeed, the maximum likelihood ancestral state reconstruction of the Ser116A substitution across 26 *Heliconius* and 1 *Eueides* species reveals this mutation arose once, in the common ancestor of *H. ismenius* and *H. numata* (supplementary fig. S2, Supplementary Material online). This known spectral tuning site may therefore be at least partially responsible for the longer-wavelength *H. erato* blue cell and suggests that *H. melpomene* also has a longer wavelength-sensitive blue cell, while *H. ismenius* may have a blue-shifted blue cell. As predicted by this spectral tuning site, intracellular recordings confirm that *H. erato* and *H. melpomene* blue cells’ peak sensitivity or *λ*_max_ = 470 nm. The *H. ismenius* blue cell's peak sensitivity is shifted 25 nm toward shorter wavelengths at *λ*_max_ = 445 nm (fig. 3*B*). Relative to *H. erato,* our intracellular recording results indicate *H. ismenius* has spectrally tuned its blue opsin—a change following the earlier loss of the UV2-expressing R1 and R2 cell in *melpomene* and silvaniform species. Although our results support this conclusion, targeted mutagenesis and *in vitro* absorbance measurements are needed to show which spectral tuning sites are definitively causal.

### Nonopsin-Based Spectral Tuning in *Heliconius*

**Fig. 4.**
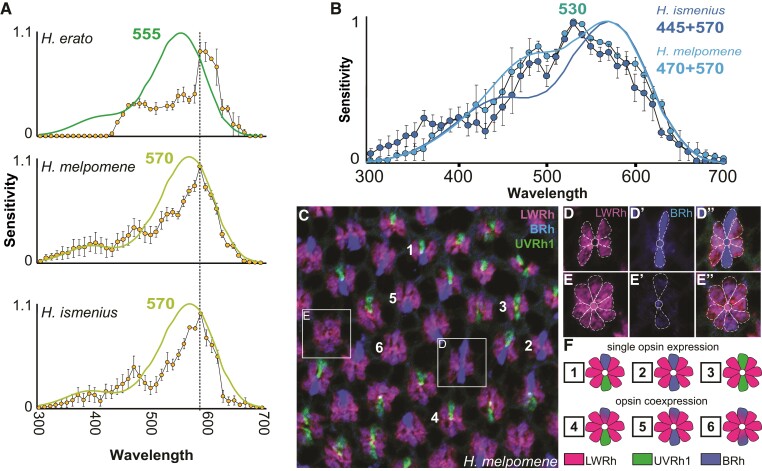
Nonopsin-amino-acid-based spectral tuning in *Heliconius* includes filtering pigments and other mechanisms. (*A*) *Heliconius erato, H. melpomene*, and *H. ismenius* all have a red-shifted cell, which has a narrower spectral sensitivity (yellow circles) (∼40–45 nm narrower) than the long-wavelength rhodopsin alone, and which peaks at 590 nm (*H. erato, n* = 2; *H. melpomene n* = 6; *H. ismenius, n* = 9). Green and chartreuse curves are rhodopsin nomograms (Stavenga et al. 1993; Stavenga 2010) for the long-wavelength rhodopsins of each species, showing the narrowed sensitivity of the cells relative to the underlying rhodopsins. Bars indicate standard errors. (*B*) In both *H. ismenius* (dark blue circles, *n* = 5) and *H. melpomene* (light blue circles, *n* = 2), we identified a cell with broad sensitivity across blue and green wavelengths, peaking at ∼530 nm. The shape of the spectral sensitivity curve of this photoreceptor does not resemble any single visual rhodopsin and its half-width at ∼155–160 nm is much wider than the ∼110 nm half-width for the 570 nm rhodopsin alone; moreover, its shape does not resemble simple combinations of blue- and green-absorbing rhodopsins in these species (*H. ismenius,* dark blue line modeling 445 and 570 nm rhodopsins; *H. melpomene,* light blue line modeling 470 and 570 nm rhodopsins). (*C–E*″) We identified LWRh opsin in R1 and R2 cells in a subset of ommatidia where BRh is also weakly expressed in *H. melpomene* (shown above), *H. ismenius, Heliconius doris, H. erato, Heliconius sara, Eueides isabella*, and *Dryas iulia* (shown in supplementary fig. S3, Supplementary Material online). Overlay of triple-stained *H. melpomene* retina with BRh (blue), LWRh (pink), and UVRh1 (green). Boxes of two example ommatidia are enlarged and cells are outlined in *D–E*″. (*F*) *Heliconius melpomene* has six known ommatidial types with respect to UVRh1 (green), BRh (blue), and LWRh (pink) expression in R1 and R2 cells, numbered in *C*.

Although most recordings in these species are well explained by opsin protein expression and known models of opsin-based absorbance spectra (Stavenga et al. 1993; Stavenga 2010), a subset of recordings are not so easily explained. One of these is the narrow-peaked yellow–orange receptor whose half-width is ∼40–45 nm narrower than the half-width of the 555 or 570 nm rhodopsin. This photoreceptor has been identified in *H. erato* despite no evidence for a corresponding orange-absorbing rhodopsin ((Bernhard et al. 1970; McCulloch et al. 2016a); see also Swihart (1972) for recordings from red-sensitive visual interneurons). Rare among insects (Briscoe and Chittka 2001; van der Kooi et al. 2021), *H. erato* can discriminate orange from red colors while another nymphalid butterfly, *Vanessa atalanta*, cannot (Zaccardi et al. 2006). Previous work in *H. erato* found a high density of red filtering pigment adjacent to the rhabdom in a subset of LWRh-expressing diagonal R5–8 cells, which absorbs blue light and shifts the cells’ overall peak sensitivity from green toward red light (fig. 1*A* and *B*) (Zaccardi et al. 2006). Like *H. erato,* both *H. melpomene* and *H. ismenius* have a yellow–orange-sensitive cell, and in all three species, this photoreceptor cell peaks at *λ*_max_ = 590 nm (fig. 4*A*). Spectral tuning of this photoreceptor has proceeded through a filtering mechanism rather than through gene duplication and molecular evolution of opsin, as seen in this cell subtype's narrowed sensitivity spectrum (fig. 4*A*).

We found another photoreceptor subtype in males and females of both *H. ismenius* and *H. melpomene* characterized by an unusually broad spectral sensitivity in the blue–green range (fig. 4*B*). The half-width of the broadband cell at ∼155–160 nm is much wider than the ∼110 nm half-width for the 570 nm rhodopsin template alone. We hypothesized that the unusually broad sensitivity might be due to opsin co-expression. Alternatively, the dip in sensitivity between this photoreceptor's peak wavelength at 530 and 400 nm suggests self-screening or filtering over these wavelengths. Using an expanded combination of antibodies, we stained and carefully observed opsin expression in *H. melpomene* compound eyes. We newly identified a subset of R1 and R2 cells that weakly express BRh and also express LWRh opsins (fig. 4*C–E*). The presence of LWRh in R1 and R2 cells is an unexpected expression domain in these ommatidia. Previously, we characterized the *H. melpomene* eye as a mosaic made up of three types of ommatidia, based on antibodies against UVRh1, UVRh2, and BRh opsins labeling R1 and R2 cells of each ommatidium (McCulloch et al. 2017). Here we identify, in addition to the previous three ommatidial types (UVRh1-UVRh1, UVRh1-BRh, BRh-BRh), three novel ommatidial types in *H. melpomene* (BRh-LWRh/BRh-LWRh, BRh-LWRh/BRh, BRh-LWRh/UVRh1) (fig. 4*D–F*). This brings the retinal mosaic of *H. melpomene* up to six known ommatidial types. It is possible that the broadband blue green-sensitive photoreceptor cell we measured with intracellular recordings is the same cell type as the one we observe which co-expresses the LWRh and BRh opsins. The broadband cell's sensitivity is not easily modeled by a blue and green rhodopsin combination alone with both broad expression and a dip in shorter wavelength sensitivity (fig. 4*D*). We suggest that these broadband photoreceptors may contain an as-yet-unidentified short-wavelength bandpass filter distinct from the red filtering pigment. The broadband cell's sensitivity could be achieved through a combination of filtering effects and coexpresssion of BRh and LWRh as discovered here, or may represent yet another distinct photoreceptor found in a subset of ommatidia.

We found the BRh and LWRh co-expressing photoreceptor cell types in both *H. ismenius* and *H. melpomene*, so we checked other representatives of major clades in *Heliconius* as well as outgroup genera *Eueides* and *Dryas.* In every species, we found instances of LWRh/BRh co-expression in R1 and 2 cells (supplementary fig. S3, Supplementary Material online). This is not due to poor specificity of our antibodies, because in all instances, we also identify adjacent ommatidia with photoreceptors expressing either blue or LW opsins but not both. This novel co-expression brings the total ommatidial types in *Heliconius sara* females up to ten (supplementary fig. S3, Supplementary Material online, for the six ommatidial types in *H. sara* females based on UVRh1, UVRh2, and BRh opsin expression alone, see fig. 1*E* and *F*, McCulloch et al. 2017; novel subtypes: BRh-LWRh/UVRh1, BRh-LWRh/UVRh2, BRh-LWRh/BRh, BRh-LWRh/BRh-LWRh). Thus we have identified additional mechanisms of spectral tuning present in other members of the tribe Heliconiini, likely as a result of a complex interaction between opsin co-expression, self-screening, and a filtering pigment. The blue and green opsin co-expression on the one hand and the newly discovered broadband blue-through-green-sensitive photoreceptor cell on the other further increases the complexity of the *Heliconius* compound eye.

## Discussion

**Fig. 5.**
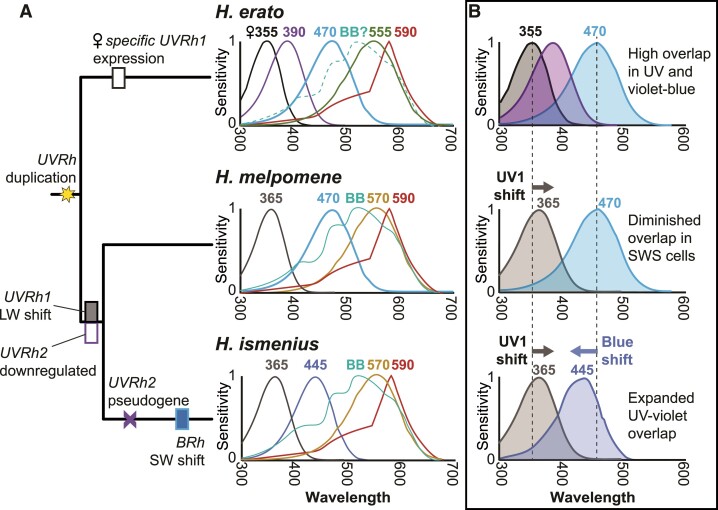
Summary of spectral sensitivities and short-wavelength photoreceptor spectral tuning across three *Heliconius* species. (*A*) Phylogeny of the three species, with molecular events associated with UV and blue opsins mapped, based on a more extensive maximum likelihood ancestral state reconstruction of additional species shown in supplementary fig. S2, Supplementary Material online. Spectral sensitivities of known photoreceptor cells in the adult compound eye of each species, averaged from both males and females. (*B*) Possible adaptive scenario for short-wavelength spectral tuning in *Heliconius. Heliconius erato* females have greater overlap in spectral sensitivities (shaded regions) in the 300–470 nm range due to the presence of two kinds of R1 and R2 UV photoreceptor cell types, UV1 (*λ*_max_ = 355 nm) and UV2 (*λ*_max_ = 390 nm), as well as the 470 nm blue cell, compared with male *H. erato* with only UV2 and blue, suggesting good female color discrimination in wavelengths where the overlap is high. In color discrimination tests, female *H. erato* can distinguish between 380 and 390 nm light, but male *H. erato* cannot (Finkbeiner and Briscoe 2021). In *H. melpomene* and *H. ismenius*, the UV2 cell is absent, and UV1 has shifted 10 nm closer to blue wavelengths. In color discrimination tests, *H. melpomene,* like *H. erato* males, cannot distinguish between 380 and 390 nm light (Finkbeiner and Briscoe 2021). In *H. melpomene*, the spectral sensitivity of the blue cell is the same as in *H. erato* resulting in decreased overlap of sensitivities in UV-to-blue wavelengths in *H. melpomene* relative to *H. erato*. In *H. ismenius*, the blue cell has subsequently shifted toward the UV, and both cells now contribute to increased overlap (relative to *H. melpomene*) in short wavelengths. For relative timing of each of these events, based on maximum likelihood reconstruction of UV and blue opsin characters mapped on a larger phylogeny, see supplementary fig. S2, Supplementary Material online. Numbers above each curve represent *λ*_max_ values.

This study compares photoreceptor spectral sensitivities in two species of *Heliconius* that have lost the UV2 but retained the UV1 receptor subtype, relative to *H. erato* which has two UV photoreceptor subtypes in R1 and R2 cells. How *H. ismenius* and *H. melpomene* visual systems have shifted relative to *H. erato* is summarized in fig. 5*A* (full data, supplementary figs. S1 and S2, Supplementary Material online). For both of these species, we count at least five spectrally distinct photoreceptor cell types. We find that both species have tuned their photoreceptors across the UV to green spectral range relative to *H. erato*. This suggests multiple adaptive shifts in color vision in *Heliconius* species, particularly in short wavelengths, after gain and subsequent loss of the UV2 cell.

### Evolutionary Implications for Loss of the UV2 Cell and Opsin Spectral Tuning

Our data paint a complex picture of evolutionary shifts in visual physiology after the loss of the UV2 cell (fig. 5*A* and *B*). We concluded from previous molecular and behavioral data that color vision in the near-UV/violet is important for female *H. erato* butterflies (Finkbeiner and Briscoe 2021). In contrast, *H. melpomene* appears to be actively downregulating *UVRh2* expression, leading to diminished UV color vision, through at least two concerted mechanisms: chromatin reorganization and post-transcriptional regulation, suggesting that this is not a random loss of expression, but rather than the loss of the UV2 cell is in some way adaptive. It is possible that maintaining two UV receptors and their associated neural wiring may be too energetically burdensome for the small benefit in UV color discrimination it imbues on the eye. Perhaps for female *H. erato,* the benefits outweigh the costs, but in *H. erato* males and in both sexes of the *melpomene/*silvaniform lineages, a single UV receptor will do. This could explain the complex pattern of independent loss of expression of either UV1 or UV2 depending on species and sex across *Heliconius* lineages. In the absence of the UV2 receptor, shifting the peak sensitivity of the UV1 cell to longer wavelengths as observed in *H. melpomene* and *H. ismenius* could give both species a boost in color discrimination in this range due to greater overlap between the UV and blue photoreceptors (fig. 5*B*). The observed shift in peak sensitivity of the *H. ismenius* blue cell to shorter wavelengths should further enhance this.

Shifting natural and sexual selective pressures could explain the species- and sex-specific differences in *Heliconius* visual systems; however, adaptive explanations are not clear for all cell sensitivities identified in this paper. Particularly, the green cell has a long-wavelength shift of ∼10–15 nm in *H. ismenius* and *H. melpomene* compared with *H. erato.* This shift could be due to shifting salience of color signals as speciation has occurred, like mimetic wing patterns or host plants for nectar, pollen, and oviposition, independent of UV2 cell subtype loss. Alternatively, the loss of UV color vision could reduce the sensitivity to UV spectral signals in the wing or on host plants, thus selecting for shifts in sensitivity to better detect other components of signal spectra. Establishing how the competing regimes of drift, natural, and sexual selection interplay to result in this regulatory and physiological diversity is challenging. More behavioral color vision and mate choice tests are necessary for these and other species to better understand the ways in which the diversity of spectral receptors in *Heliconius* are used.

### Opsin Genomic Analysis

Our *H. melpomene* ATAC-seq data corroborate our opsin expression and spectral sensitivity data for this species, where the chromatin is more open and thus actively transcribed at the *UVRh1* locus relative to the *UVRh2* locus. Peak analysis has allowed for the identification of candidate transcription factors that could be maintaining UV1 cell-type identity, although these are not all classical eye-related transcription factors. It has been previously shown that Pax6/eyeless binds directly to the *rhodopsin* (*rh*) promoter in *Drosophila* (Sheng et al. 1997; Papatsenko et al. 2001). A search for the consensus Pax6 *rh* binding site motif TAATYNRATTN through the *H. melpomene UVRh1* locus in the genome identifies only one motif not near any peaks in the first intron of *UVRh1*. We also identify a Pax6-binding site in the promoter of *UVRh2*, but this is not near a peak of open chromatin. We did not expect all eye-related developmental transcription factors to be bound to peaks in adult tissue. Many photoreceptor differentiation and determination genes may not be involved at all in adult maintenance of photoreceptor cells and adult function of developmental eye-related genes is not well studied. Other mechanisms of transcription factor binding such as cofactor binding sites or Pax6 paired domain—instead of homeodomain-binding—may be involved. It is therefore of note that Optix (Six3/6) and Shaven (Pax2/5/8) binding sites are bound in peak 1, suggesting combinatorial expression of particular retinal determination network paralogs could generate distinct regulatory modules in both development and adult maintenance of *UVRh1*-expressing photoreceptor cells. Optix is additionally co-opted into wing patterning phenotypes across the tribe Heliconiini, and there is a genetic linkage between mate choice and color pattern in *Heliconius* (Jiggins et al. 2001; Kronforst et al. 2007; Martin et al. 2014; Merrill et al. 2019). Optix is an interesting candidate for future study of visual system regulation across species as well.

### Filtering Pigments and Co-expression as Mechanisms for Spectral Tuning in *Heliconius*

Filtering of light appears to play a large role in the spectral richness of the *Heliconius* compound eye. The yellow–orange-sensitive photoreceptor, shared across all three species, displays a narrower, steeper peak than other cell types. This yellow–orange sensitivity spectrum does not match a typical rhodopsin-based photoreceptor template, which predicts a much wider half-width of ∼110 nm versus the observed half-width of ∼65–75 nm (fig. 4*A*) (Stavenga et al. 1993). The likely candidate for generating this cell type in all three species is the red filtering pigment identified in the diagonal R5–8 photoreceptor cells of the *H. erato* eye (see fig. 6C in Zaccardi et al. 2006), although the molecular composition of this pigment has yet to be fully characterized. Langer and Struwe (1972) noted the presence of an ommochrome-based pigment with peak absorbance near 560 nm in the eyes of *H. erato, H. sara,* and *H. numata*. This evidence, the location of the pigment in the eye, intracellular recordings, and behavior (Zaccardi et al. 2006) all support that this is the likely filtering pigment in *Heliconius*, but additional experiments are needed to confirm its identity and function.

We found physiological evidence for a previously unknown broadband cell in *Heliconius.* The shape of the *Heliconius* broadband cell resembles one of the long-wavelength photoreceptor classes recorded from the eye of the nymphalid, *Polygonia c-aureum* (Kinoshita et al. 1997). We also found immunohistochemical evidence for the co-expression of LWRh and BRh in a subset of R1 and R2 photoreceptor cells. This raises the question as to whether the broadband cell and the LWRh and BRh co-expressing cells are the same class of photoreceptor cell or different classes. The difficulty of fitting mixed blue and green rhodopsin templates to these curves, without adding other variables, such as self-screening, filtering by adjacent rhabdomeres, and/or filtering by a photostable pigment, makes it hard to arrive at any conclusion(Snyder et al. 1973; Nagloo et al. 2020). A separate, physiological study of *H. erato* also found evidence for a green-sensitive R1/R2 cell with a hyperpolarizing response to red light (Belušič et al. 2021). Whether or not they are the same cell, our protein expression data add to growing evidence that opsin co-expression may be a common way of modulating spectral sensitivity in animal photoreceptor cells (Applebury et al. 2000; Arikawa et al. 2003; Sison-Mangus et al. 2006; Ogawa et al. 2012; Dalton et al. 2014, 2017). A broadband spectral cell type has been identified in *Papilio xuthus* via two LWRh opsins co-expressed in the R5–8 cells of a particular ommatidial type, together with a 3-hydroxyretinol UV-fluorescing pigment in the distal rhabdom (Arikawa et al. 2003). An experimental paradigm using monochromatic light (Koshitaka et al. 2008) could be used to rule out or rule in the use of the broadband receptor in *Heliconius* color vision. In addition to opsin co-expression, the expression of LWRh, which is typically reserved for R3–8 cells, is found in R1 and R2 cells at least in the tribe Heliconiini (supplementary fig. S3, Supplementary Material online). This is a novel and significant finding because it represents a regulatory switch in an otherwise highly constrained developmental program in most insects. Exceptions to this strict R1 and R2 and R3–8 regulatory boundary do exist, such as in the dorsal rim area of monarch butterflies, where all R1–8 cells express UV (Sauman et al. 2005). This is the first time we observe LWRh protein expression in R1 and R2 cells in any nymphalid.

## Conclusion

Physiological comparison of photoreceptors in the eyes of three related species shows that ongoing visual system evolution appears to be occurring in a compensatory manner in *Heliconius*. We posit that after the loss of the UV2 photoreceptor in *H. ismenius* and *H. melpomene,* spectral tuning of first the UV1 rhodopsin in these two species, followed by the blue rhodopsin in *H. ismenius* made up for their loss of UV color discrimination ability relative to the *H. erato* visual system. We show that a variety of mechanisms of opsin gene expression regulation, filtering, and spectral tuning are employed within single species in this genus, generating a diversity of spectrally distinct photoreceptors not limited by the number of opsins in the genome.

## Materials and Methods

### Animals

We obtained *H. ismenius telchinia* and *H. melpomene rosina* pupae from The Butterfly Farm—Costa Rica Entomological Supply. After eclosion, butterflies were housed for at least 1 day in a humidified chamber and were fed a diluted honey solution daily before recording. Animals were killed after recording by rapidly severing the head and crushing the thorax.

### *Heliconius melpomene* ATAC-seq

After sacrificing, the heads of one male and one female recently eclosed *H. melpomene* were placed in a small Petri dish with Butterfly Ringer's solution (35 mM NaCl, 36 mM KCl, 12 mM CaCl_2_, 16 mM MgCl_2_, 274 mM glucose, and 5 mM Tris–HCl, pH 7.5) to dissect and separate photoreceptor cells from brain tissue. Dissected brain and photoreceptors were placed in separate 1.7 ml microcentrifuge tubes containing 500 μl Ringer's. The tubes were centrifuged at 500 × g for 5 min at 4 °C. Tissue was washed with 500 μl 1× PBS buffer and centrifuged at 500 × g for 5 min at 4 °C. We added 100 μl of cold lysis buffer (10 mM Tris–HCl, pH 7.4, 10 nM NaCl, 3 mM MgCl_2_, 0.1% IGEPAL CA-630; Sigma-Aldrich, St Louis, MO, USA) and ground the cells with a pestle. We transferred the ground mixture into a Nucleospin filter (Fisher Scientific, Pittsburgh, PA, USA) and centrifuged the column at 500 × g for 10 min at 4°C. We did a transposition reaction using 25 μl 2× TD buffer, 2.5 μl Tn5 Transposase, and 22.5 μl Nuclease Free water. The reaction was incubated at 37 °C for 30 min and purified using a MinElute Reaction Cleanup Kit (Qiagen, Germantown, MD, USA). The PCR reaction consisted of 30 μl transposed DNA, 2.5 μl each of 25 μM customized Nextera PCR primer 1 and primer 2, and 30 μl Phusion DNA polymerase mix (New England Biolabs). The reaction was incubated at 72°C 5 min, 98°C 30 s, 9 cycles of (98°C 30 s, 63°C 30 s, 72°C 1 min), hold at 4°C. The product was run on a gel and 100–500 bp fragments were size selected; recovered DNA was purified using a MinElute PCR Purification Kit (Qiagen). Two replicates for each sex and tissue type (male brain, male photoreceptors, female brain, female photoreceptors) of *H. melpomene* were sequenced using Illumina paired-end 43 bp reads on a NextSeq 500 sequencer. After sequencing, adapters and low-quality base pairs were trimmed using Trimmomatic v. 0.35 with the following parameters: PE [Read1.fastq] [Read2.fastq] paired_Read1.fastq.gz unmated_Read1.fastq.gz paired_Read2.fastq.gz unmated_ Read2.fastq.gz ILLUMINACLIP:NexteraPE-PE.fa:2:30:8:4:true LEADING:20 TRAILING:20 SLIDINGWINDOW:4:17 MINLEN:30. Paired reads were mapped to the Hmel2.5 genome (unpublished; see also Davey et al. 2016; lepbase.org) using bwa aln in bwa v. 0.7.8. Duplicate reads were removed using Picard tools v. 1.96 (http://broadinstitute.github.io/picard) with the following parameters: MarkDuplicates INPUT = input.bam OUTPUT = output.bam METRICS_FILE = metrix.txt REMOVE_DUPLICATES = true VALIDATION_STRINGENCY = LENIENT. Peaks were called using callpeak in MACS2 v. 2.2.7.1 with default parameters. Identified peaks in any of the samples near the *UVRh1* and *UVRh2* loci were scanned using the JASPAR database for potential transcription factor binding sites, using a 95% threshold level. Peaks across tissue replicates were merged using bedtools (v. 2.25.0) intersect to find consensus peaks and bam files were merged using samtools (v. 1.9) merge. Analysis of bound and unbound motifs was done using the pipeline in TOBIAS (Bentsen et al. 2020). After merging bed and bam files, we used ATACorrect to remove Tn5 bias, ScoreBigwig to calculate footprint scores, and BINDetect to determine bound positions. ATAC-seq data were visualized in IGV.

### Intracellular Recording

Detailed written and video protocols are published elsewhere (McCulloch et al. 2016a, 2016b) and are described briefly here. Only one cell from one individual was used for each biological replicate. The sex of the individual was determined then it was affixed inside a small humidified plastic tube using hot wax. The tube was mounted on a stage and a 0.125 mm diameter indifferent silver electrode was inserted into the head via the mouthparts. A small hole (∼10 ommatidia in diameter) was cut in the cornea using a thin razor blade and sealed with Vaseline to prevent desiccation. We used an Oriel Xenon Arc lamp (Irvine, CA, USA) as a light source. The light was passed through a condenser lens assembly (Model 60006, Newport, Irvine, CA, USA), a convex silica lens (SPX055, Newport), a neutral density (ND) filter wheel (0–3.5 optical density), 10 nm bandwidth spectral interference filters (40 filters, spanning 300–700 nm, Edmund Optics, Barrington, NJ, USA), a concave silica lens (Newport SPC034), a shutter with a drive unit (100-2B, Uniblitz, Rochester, NY, USA), and a collimating beam probe (77644 Newport), into an attached UV-transmitting 600 μm diameter fiberoptic cable (78367 Oriel), on an optical rail. Photoreceptors were recorded intracellularly with sharp borosilicate capillary microelectrodes filled with 3 M KCl (∼100 MΩ tip resistance). To be analyzed, the recording had to be stable throughout data collection, that is, no change in resting potential, at least 10:1 signal to noise ratio, and large depolarizing responses (at least ∼50 mV response amplitude). Responses of narrow-band spectral flashes of 50 ms were recorded, at 0.5 s time intervals and covering the spectrum from 300 to 700 nm in steps of 10 nm. Intensity response curves were recorded from 3.5 to 0 optical density before and after an experiment to confirm no change in the recording throughout.

Spectral sensitivities of cells were derived as follows. The responses to white light at each ND filter step were used to create a response–log intensity (*V*log*I*) curve. The *V*log*I* data were used to estimate parameters for the Naka–Rushton equation: *V/V*_max_ = *I^n^*/(*I^n^ + K^n^*), where *V* is the amplitude of a given response; *V*_max_ is the maximum response amplitude; *I* is the intensity of the stimulus for the given response, *V*; *K* is the intensity of the stimulus that elicits half of *V*max; and *n* is the exponential slope of the function (Naka and Rushton 1966; Aylward 1989; McCulloch et al. 2016a, 2016b). Correction factors were calculated to approximate constant photon flux over all filters from 300 to 700 nm to account for differences in total photon flux for each interference filter. Corrected intensities were divided by the maximum response intensity for each cell to calculate relative spectral sensitivity. Photoreceptors were classified by peak sensitivity and shape of the spectral sensitivity curve and replicates were averaged and standard error was calculated for the sensitivity at each wavelength. To estimate peak sensitivities, we used least-squares regression to fit rhodopsin templates to our data (supplementary table S4, Supplementary Material online) (Stavenga 2010). All cells and sensitivities are found in supplementary table S3 and fig. S1, Supplementary Material online.

### Cryosectioning and Immunohistochemistry

Detailed methods and antibody generation are described in McCulloch et al. (2016a, 2017). Briefly, freshly severed butterfly eyes were immediately fixed in 4% paraformaldehyde (Electron Microscopy Sciences, Hatfield, PA, USA) in 1× phosphate-buffered saline (PBS) for 30 min at room temperature. Eyes were then step-wise sucrose-protected up to 30% in PBS. Each eye was placed in Tissue Tek O.C.T. compound (VWR, Radnor, PA, USA), frozen at −20°C, and sectioned at 14 μm thickness on a Microm HM 500 OM microtome cryostat (Fisher Scientific, Pittsburgh, PA, USA). Slides were dried overnight at room temperature. Dry slides were placed in 100% ice-cold acetone for 5 min, then washed 3×10 min in PBS. Slides were then placed in 0.5% sodium dodecyl sulfate in PBS for 5 min. Each slide was blocked for 1 h at room temperature using 8% (v/v) normal donkey serum and normal goat serum, and 0.3% Triton X-100 in PBS. Slides were incubated with 1:15 guinea pig anti-UVRh1, 1:15 rat anti-BRh, and 1:15 rabbit anti-LWRh antibody in a blocking solution overnight at 4 °C. Slides were washed 3 × 10 min in PBS and then incubated with 1:1000 goat anti-rat Alexafluor 488 and 1:500 donkey anti-rabbit Alexafluor 555, and 1:250 goat anti-guinea pig Alexafluor 633 (Life Technologies) in blocking solution for 2 h at room temperature. Slides were washed again 3 × 10 min in PBS. Images were taken using a Zeiss LSM 700 confocal microscope under a 20× objective, in the UC Irvine Optical Core Facility. Stains were pseudocolored, and contrast and brightness were adjusted for clarity using Adobe Photoshop CS4 and Fiji (Schindelin et al. 2012).

### Character Mapping

*Heliconius* characters were mapped on the modified *Heliconius* species phylogeny for which we have sequence data from McCulloch et al. (2017) using Mesquite v.3.10. Ancestral character likelihood analysis was performed in Mesquite using binary character states. For *H. numata* and *H. hecale UVRh2* mutations for which we have evidence for both intact and pseudogenes in different individuals, we coded these as having the intact gene locus present.

## Supplementary Material

msac067_Supplementary_Data

## Data Availability

All the data of our study are publicly available at EMBL-EBI ArrayExpress under accession code E-MTAB-6342 and E-MTAB-10684 and at NCBI Genbank under accession codes: EU358780, MF035499, MF035575, MF035650, MF035609, MF035638, MF035666, AY587902, AY587903, AY918902, AB208675 and AB208674.
