# Supplementary material for: Multiple Mechanisms of Photoreceptor Spectral Tuning in Heliconius Butterflies
Source: Mol Biol Evol. 2022 Mar 28;39(4):msac067. doi: 10.1093/molbev/msac067 (PMC9048915; doi:10.1093/molbev/msac067)
Supplement: msac067_Supplementary_Data [file msac067_supplementary_data.pdf]

Supplementary Data

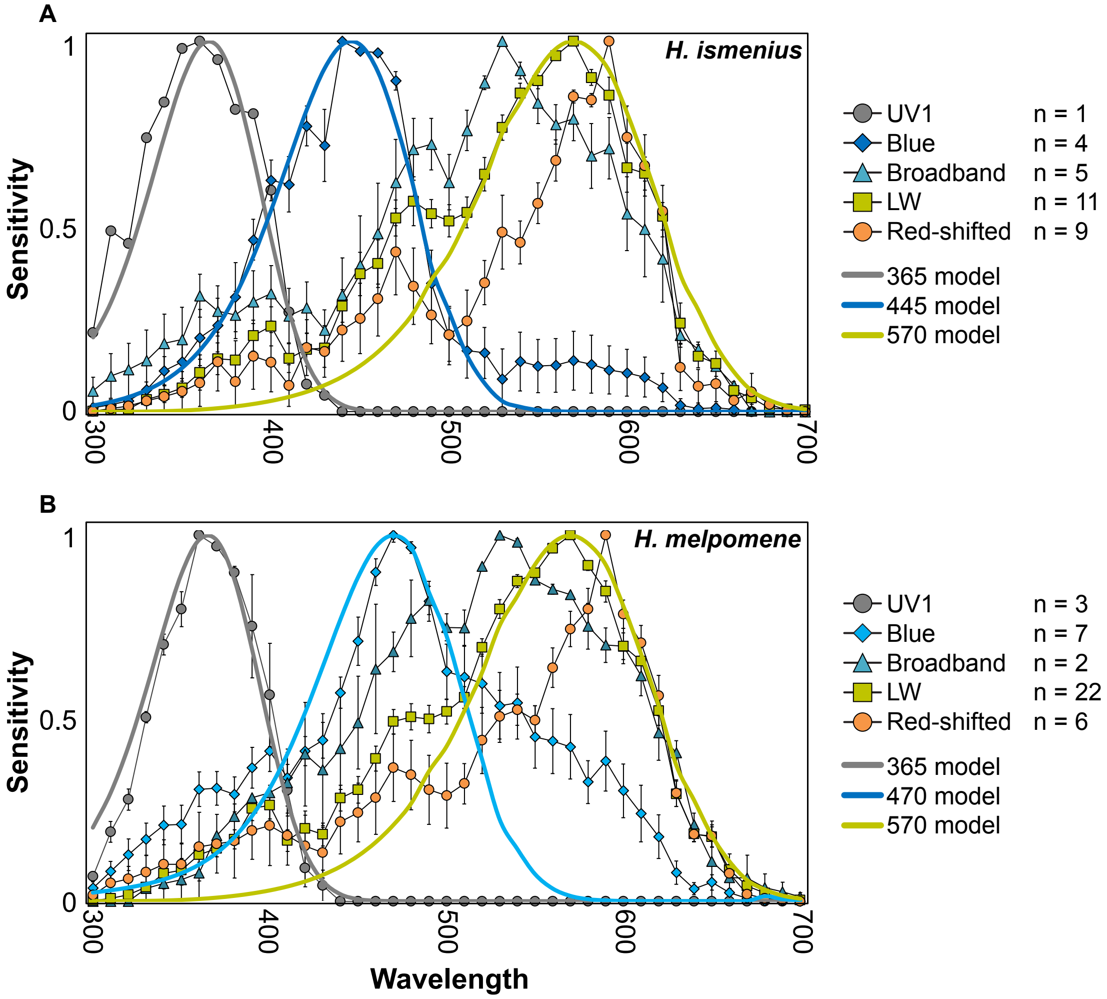

Figure S1. Averaged spectral sensitivities in *H. ismenius* and *H. melpomene*. All recordings used for **A)** *H. ismenius* and **B)** *H. melpomene* were averaged and presented here with standard error bars. Rhodopsin absorbance spectra are also shown for cells that appear to express a single kind of opsin. For each photoreceptor cell type, the number (n) of individual cells recorded from are listed.

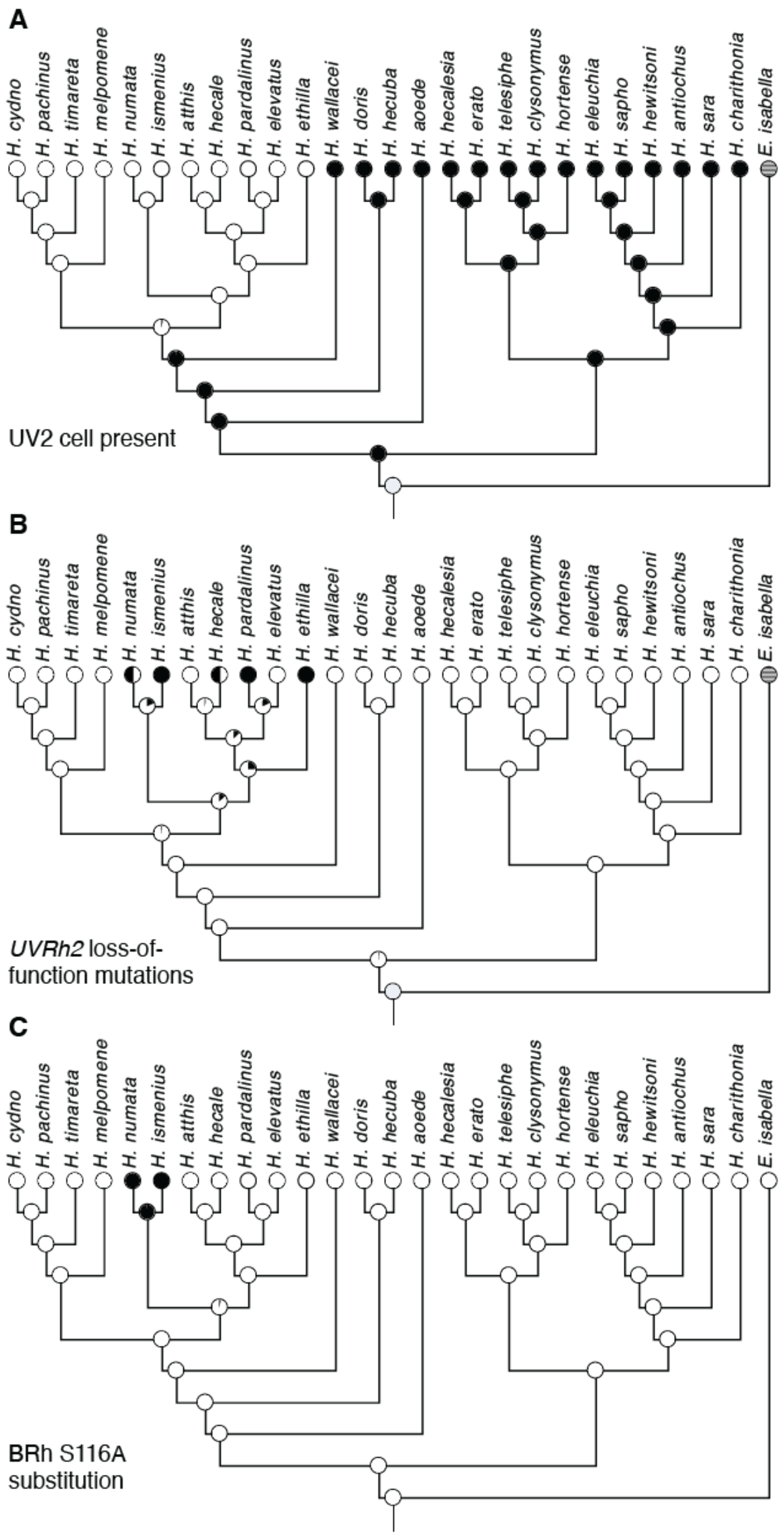
A
H. cydno
H. pachinus
H. timareta
H. melpomene
H. numata
H. ismenius
H. atthis
H. hecale
H. pardalinus
H. elevatus
H. ethilla
H. wallacei
H. doris
H. hecuba
H. aoede
H. hecalesia
H. erato
H. telesiphe
H. clysonymus
H. hortense
H. eleuchia
H. sapho
H. hewitsoni
H. antiochus
H. sara
H. charithonia
E. isabella
UV2 cell present
B
UVRh2 loss-of-function mutations
C
BRh S116A substitution

Figure S2. Ancestral state reconstruction of UVRh and BRh character mapping. Maximal likelihood analysis was run in Mesquite using species we have previously sequenced and/or immunolabeled. **A)** Previously known expression of UVRh2 protein (black) is mapped and shows a loss of expression (white) at the base of the clade including *H. melpomene* and *H. ismenius. E. isabella* is shaded out because this species diverged before the *UVRh* duplication event. **B)** The presence of any loss-of-function mutations known in the *UVRh2* gene locus were mapped onto the phylogeny (black). *H. numata* and *H. hecale* were found to have both intact and non-functional copies of *UVRh2,* depending on the individual sequenced (black and white, coded as intact gene present for likelihood analysis). Ancestral state reconstruction suggests loss-of-function is occurring multiple times in parallel within this clade. **C)** The presence of the S116A substitution is mapped (black), which has been shown , by site-directed mutagenesis of the blue-absorbing rhodopsins in other butterflies, to be a spectral tuning site. Of all *Heliconius* species sequenced, this has evolved only once, recently, *within* the silvaniform clade, well after loss of the UVRh2 cell.

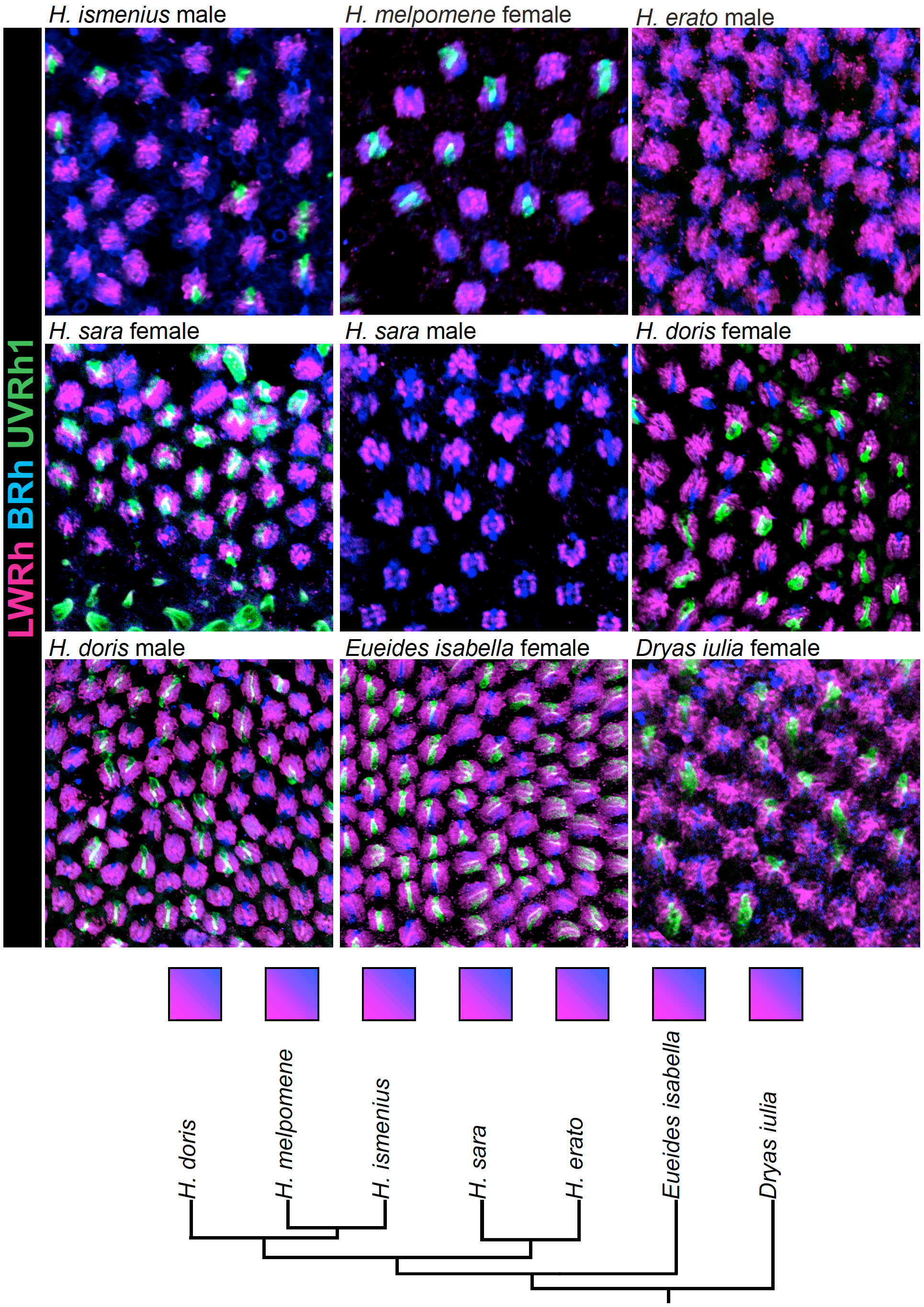

Figure S3. Antibody stains for LWRh and BRh show opsin coexpression in *Heliconius* species and outgroups. Triple labelling of photoreceptor cells in the adult compound eyes of *H. ismenius, H. melpomene, H. erato, H. sara, H. doris, Eueides isabella* and *Dryas iulia* using anti-LWRh (pink), anti-BRh (blue) and anti-UVRh1 (green) antibodies reveals coexpression of LWRh and BRh in every major clade within *Heliconius* as well as in the outgroup genera *Eueides and Dryas.*

Table S1. ATAC-seq samples and statistics.

Sample	Specimen	Sex	Tissue	Reads	Paired	% paired	Reads Aligned	% mapped	% properly paired	Total Peaks
HMP516b	HMP516	F	brain	31518059	30372185	96.36	36740592	100	91.68	13275
HMP516e	HMP516	F	photoreceptors	36448289	35180835	95.52	42110559	100	87.79	9009
HMP517b	HMP517	M	brain	36025856	34750866	96.46	41873107	100	91.76	22890
HMP517e	HMP517	M	photoreceptors	55863519	53884930	96.46	65897894	100	90.39	6304
HMP540b	HMP540	F	brain	41305222	39817558	96.4	45787536	100	91.53	4101
HMP540e	HMP540	F	photoreceptors	30515084	29270537	95.92	36601722	100	92.57	4781
HMP541b	HMP541	M	brain	16349872	15661333	95.79	20077349	100	91.39	4457
HMP541e	HMP541	M	photoreceptors	30691619	29773599	97.01	37971102	100	93.02	5455

Table S2. TOBIAS footprinting scores for eye and brain *UVRh1* and *UVRh2* loci.

(separate table)

Table S3. Species, sex, and number of recorded cells used in this study.

Species	Sex	Cell Peak					
		365	445	470	530	570	590
H. ismenius	female	1	1	-	3	6	5
H. ismenius	male	0	3	-	2	5	4
H. melpomene	female	0	-	1	1	8	4
H. melpomene	male	3	-	6	2	14	2

Table S4. Least squares regression fits summary.

H. ismenius						
	Estimate (λmax)	Std. Error	t value	Pr(>|t|)	Residual St. Error	d.f.
**UV1**	**365.42**	1.252	291.9	<2e-16	0.06095	15
**B**	**447.78**	2.298	194.9	<2e-16	0.08371	26
**LW**	**564.73**	1.083	521.4	<2e-16	0.04174	14
H. melpomene						
	Estimate (λmax)	Std. Error	t value	Pr(>|t|)	Residual St. Error	d.f.
**UV1**	**367.29**	0.4457	824.1	<2e-16	0.02072	35
**B**	**471.36**	2.839	166	3.22E-12	0.0892	6
**LW**	**565.64**	0.9007	628	<2e-16	0.03174	14
